# Three new species of *Diplotaxis* Kirby from Guatemala and Mexico (Coleoptera, Scarabaeidae, Melolonthinae), with a key to the species of the *trapezifera* group

**DOI:** 10.3897/zookeys.993.49434

**Published:** 2020-11-16

**Authors:** Leonardo Delgado, Víctor Hugo Toledo-Hernández

**Affiliations:** 1 Centro de Investigación en Biodiversidad y Conservación, Universidad Autónoma del Estado de Morelos, Avenida Universidad No. 1001, Col. Chamilpa, 62209 Cuernavaca, Morelos, México Universidad Autónoma del Estado de Morelos Cuernavaca Mexico; 2 Instituto de Ecología, A. C. Carretera Antigua a Coatepec 351, 91070 Xalapa, Veracruz, México Instituto de Ecología Veracruz Mexico

**Keywords:** Cloud forests, Description, Diplotaxini, Insecta, Mesoamerica, taxonomy

## Abstract

Three new species of *Diplotaxis* Kirby are described and illustrated, *D. balam***sp. nov.** from Guatemala, and *D. chiapasensis***sp. nov.** and *D. complanatis***sp. nov.** from Mexico. The new species have a flattened body and are included in the *trapezifera* species group. An updated key to the *trapezifera* species group is given.

## Introduction

The American genus *Diplotaxis* Kirby is the third most diverse genus among the New World Melolonthinae and the second largest Diplotaxini genus worldwide (Bezdek 2004; Evans and Smith 2009). This genus contains 237 described species distributed from Canada through the West Indies to Brazil. Mexico has the highest diversity with 181 species, followed by the United States with 105 species (Vaurie 1958, 1960; McCleve 1993; Delgado and Mora-Aguilar 2012; Cherman et al. 2016). The species of this genus are arranged in 37 species-groups, with nine species unassigned to any group (Vaurie 1958, 1960; Delgado and Capistrán 1992; Davidson and Davidson 2006).

In this work we describe three new species of *Diplotaxis*, which share with *D. xalapensis* Delgado & Capistrán, 1992 the following characters: body dorsoventrally flattened, clypeus setose, pronotum and elytra glabrous or nearly so. These species are diagnosed and included in the key to the species of the *trapezifera* group below.

## Materials and methods

Morphological structures were studied using a Zeiss Stemi SV-6 stereomicroscope. Photographs were taken with a Nikon SMZ25 stereomicroscope and a DS-Fi2 camera and images were processed with the NIS-Elements software. Measurements were taken with an ocular micrometer. The length of the beetles was measured from the apex of the clypeus to the apex of the pygidium, whereas the width was measured across the maximum width of the elytra. Morphological terminology follows that of Vaurie (1958, 1960).

Abbreviations for collections cited in this work are as follows: **UVGC** – Colección Entomológica de la Universidad del Valle de Guatemala (Guatemala, Guatemala), **CNIN** – Colección Nacional de Insectos de la Universidad Nacional Autónoma de México, (Mexico City), **ECO-SC** – Colección Entomológica de El Colegio de la Frontera Sur (Chiapas, Mexico), **IEXA** – Colección Entomológica del Instituto de Ecología, A. C. (Veracruz, Mexico), **SMC** – Scott McCleve private collection (Arizona, USA), and **LLDC** – Leonardo Delgado private collection (Veracruz, Mexico).

## Results

### Diplotaxis balam sp. nov.

Taxon classificationAnimaliaBrassicalesBrassicaceae

898C96D1-1C00-556E-B175-02B347ECF393

http://zoobank.org/75CDF203-6811-4A1E-8D85-40547CEC4FBD

Figs 1–5

#### Material examined.

***Holotype*** male,“Guatemala: Zacapa, arriba de La Unión, 16-III-1996, Alt. 1,550 m, bosque nuboso, J. C. Schuster col.” (UVGC). ***Paratype*** female, same data as holotype (LLDC).

#### Diagnosis.

**Figures 1–5.**
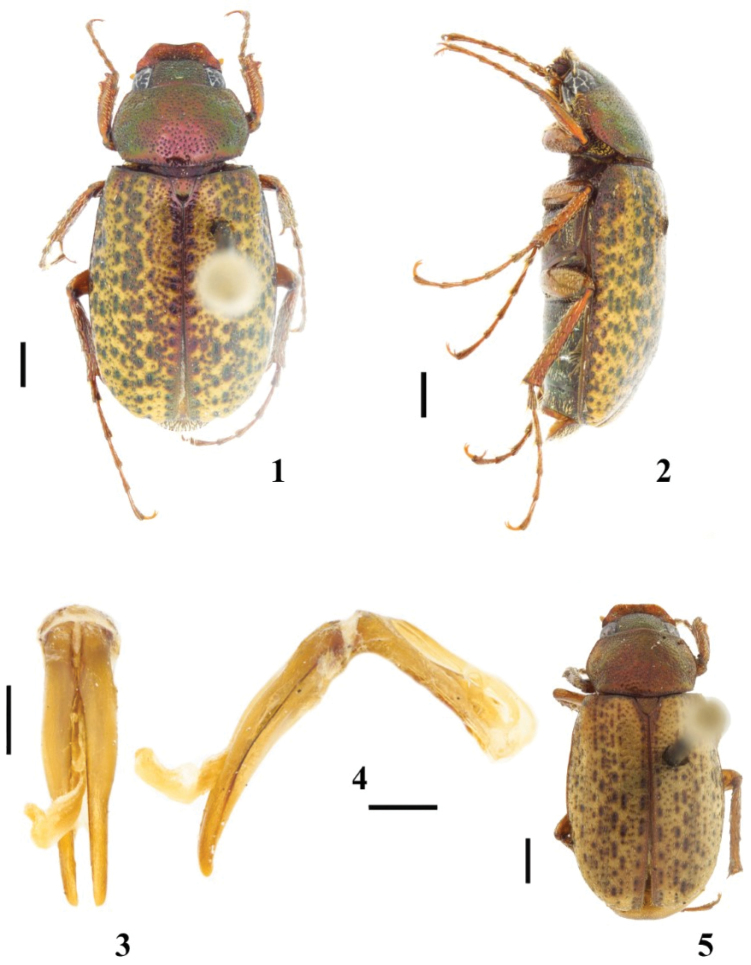
*Diplotaxis balam* sp. nov. **1** male habitus, dorsal view **2** male habitus, lateral view **3** aedeagus, dorsal view **4** aedeagus, lateral view **5** female habitus. Scale bars: 1 mm (**1, 2, 5**); 0.5 mm (**3, 4**).

This species is easily recognized by the color of the elytra (Figs 1–2, 5): ground color yellow with black, irregular foveae distributed throughout entire surface. No other described species of this genus shows this color pattern.

#### Description.

***Holotype*. Male** (Figs 1–4). Length 8.55 mm; width 4.21 mm. Body elongate and dorsoventrally flattened. Clypeus red with a metallic green tinge, frons and pronotum metallic green, elytra yellow and black with irregular foveae throughout entire surface, venter metallic green, legs tan with a weak metallic green tinge.

***Head*.** Clypeus trapezoidal in shape, 0.4 length of frons, apex broadly sinuated with anterior angles rounded, sides indented in front of eyes; clypeal surface concave, rugose, with distinct setae; frontoclypeal suture absent; frons flat, gradually declivous to clypeus, with coarse, dense punctures; transverse eye diameter 0.33 interocular width; antennae 10-segmented; labrum flat, flush with, and 0.20 times longer than reflexed underside face of clypeus, surface with dense punctures; mandibles slender in frontal view; mentum slightly convex, with weak anterior declivity marked by a suture; last article of maxillary palps not impressed dorsally.

***Pronotum*.** Hexagonal in shape; with anterior angles acute, lateral margins angled and situated behind middle, posterior angles obtuse; pronotal surface almost evenly convex, with three lateral foveae on each side; pronotal punctuation regular with dense, medium-sized punctures on disc, confluent near sides; basal margin with bead not cariniform, with a continuous row of punctures; most punctures bearing a minute seta slightly longer than one puncture diameter.

***Scutellum*.** With medium-sized punctures at sides. ***Elytra***. 1.7 times longer than width, elytral surface with irregular, shallow, black foveae, most of them on the intervals; elytral striae with separated, ocellate punctures, intervals with small, moderately dense punctures; elytral punctures with setae minute but slightly longer than those of pronotum.

***Abdomen*.** Without lateral carina; propygidium without groove above of pygidium; ventrites 2–5 subequal in length, surface with setae medially and with whitish scales laterally; pygidium 1.7 times wider than long, surface with coarse, deep, setigerous punctures; setae dense, longer on apical half.

***Legs*.** Protibiae tridentate, basal tooth weak and situated in distal half; claws long, slightly curved, cleft subapically, inner rami of claws shorter than apex; tarsi longer than respective tibiae; mesotarsomere 1 slightly shorter than 2; metacoxal plate rounded and margined laterally; metafemora straight and slender; metatibial spurs slender, long, acute; metatarsomere 1 shorter than the 2 and slightly longer than longest spur.

***Genitalia*.** Basal piece shorter than parameres, which are joined on inner margin at basal fifth, moderately widened at middle, apices blunt (Figs 3–4).

**Female**. One female paratype (Fig. 5). Length 6.97 mm; width 3.53 mm. The female differs from the male in the following respects: clypeus slightly shorter; frons and vertex more convex; transverse eye diameter 0.31 interocular width; pronotum with anterior angles obtuse and lateral angles rounded; elytra 1.3 times longer than width; abdomen nearly flat; pygidium 1.6 times wider than long; tibiae broader and robust; metafemora slightly broader; inner metatibial spur wider and longer than metatarsomere 1.

#### Etymology.

The specific epithet *balam*, meaning jaguar in the Mayan language, refers to the color pattern of the elytra, similar to the skin of this feline.

#### Distribution.

This species is only known from the type locality, situated in the Sierra de Las Minas, Guatemala, near the border with Honduras (14°56'45.6"N, 89°16'40.1"W) (Fig. 17). The locality is at 1550 m altitude, covered by a cloud forest.

#### Taxonomic remarks.

The features of *D. balam* sp. nov. agree in part with those of the *trapezifera* species group [see key to species groups by Vaurie (1960)]. The group is mainly characterized by the presence of setae on the clypeal surface, the rest of the dorsum being glabrous or with minute setae only. However, *D. balam* sp. nov. (as well as *D. xalapensis* and the two new species described below) has a dorsoventrally flattened body, unlike species of the *trapezifera* group which have a convex body. *Diplotaxis balam* sp. nov. is distinguished from all other *Diplotaxis* by the unique color pattern of the elytra (Figs 1–2, 5).

### Diplotaxis chiapasensis sp. nov.

Taxon classificationAnimaliaBrassicalesBrassicaceae

6B1EB4A4-FFE2-581F-BCDC-9E35AE424597

http://zoobank.org/DE89ABE0-49DA-4BEF-A743-B0A08FAEEA7F

Figs 6–10

#### Material examined.

***Holotype*** male, “México: Chiapas, Unión Juárez, Talquián, 7-X-2002, B. Gómez y Gómez col.” (ECO-SC). ***Paratype*** female, same data as holotype (LLDC).

#### Diagnosis.

This new species is recognized by the following combination of characters: body dorsoventrally flattened; clypeus setose, rest of dorsum glabrous; dorsum with a metallic green cast; pronotum and elytra shiny, without microreticulation.

#### Description.

**Figures 6–10.**
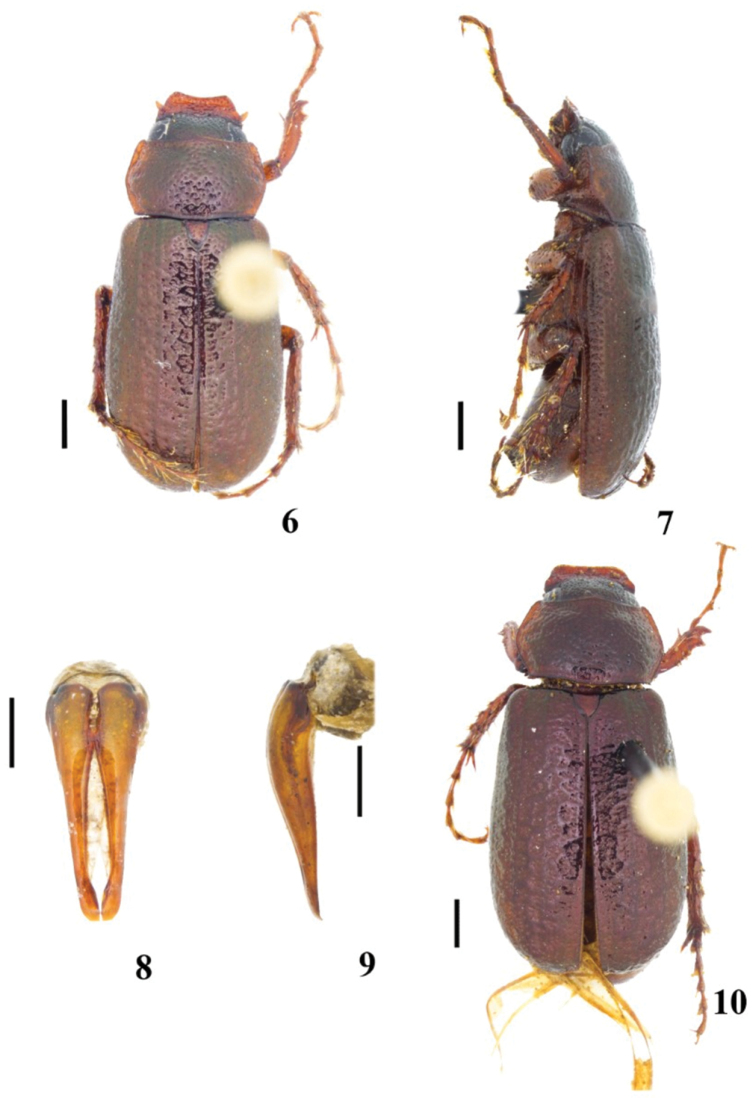
*Diplotaxis chiapasensis* sp. nov. **6** male habitus, dorsal view **7** male habitus, lateral view **8** male genitalia, frontal view **9** male genitalia, lateral view **10** female habitus. Scale bars: 1 mm (**6, 7, 10**); 0.5 mm (**8, 9**).

***Holotype*. Male** (Figs 6–9). Length 8.14 mm; width 3.63 mm. Body elongate and dorsoventrally flattened. Clypeus, sides of pronotum and scutellum reddish-brown, frons and vertex black, most of pronotum and elytra dark brown, legs and venter reddish-brown; head, pronotum and elytra with metallic green cast.

***Head*.** Clypeus subrectangular in shape, short, length equals 0.80 of that of frons and vertex combined, apex broadly sinuated, anterior angles rounded, clypeal surface with short, sparse setae; frons with anterior half gradually declivous to clypeus and slightly concave; punctuation of clypeus rugose, frons with punctures of medium size, moderately dense; transverse eye diameter 0.32 interocular width; antennae 10-segmented; labrum concave, flush with, and slightly longer than, reflexed underside of clypeus, surface with moderately dense punctures; mandibles slender in frontal view; mentum with anterior declivity marked by transverse, curved, setiferous ridge; last article of maxillary palps not impressed dorsally.

***Pronotum*.** Hexagonal in shape, anterior angles acute, lateral margins obtusely angled, posterior angles obtuse; pronotal surface almost evenly flat, reticulated, with large, ocellate punctures; lateral and basal borders narrowly beaded.

***Scutellum*.** Moderately punctate. ***Elytra***. 1.6 times longer than width, surface moderately rugose and densely punctate, punctures larger than those on pronotum; striae indistinct; marginal lateral setae scarce and minute, only present on basal fourth.

***Abdomen*.** Without lateral carina, propygidium without groove anterior to pygidium, ventrites 2–5 subequal in length, with sparse setae; pygidium 1.6 times wider than long, with confluent punctures and moderately dense setae.

***Legs*.** Protibiae tridentate, basal tooth small and situated on apical 2/5 of protibia; protarsal claws slightly curved, subapically cleft, both rami equal in length; all tarsi longer than respective tibiae, mesotarsomere 1 as long as 2; metacoxal plates truncate and margined laterally; metafemora straight and slender; metatibial spurs slender and shorter than metatarsomere 1; metatarsomere 1 shorter than 2; meso- and metatarsal claws abruptly curved, with subapical ramus large.

***Genitalia*.** Basal piece damaged, parameres joined on inner margin at basal fourth, narrowing distally to moderately widened apices (Figs 8–9).

**Female**. One female paratype (Fig. 10). Length 8.59 mm; width 3.90 mm. The female differs from the male in the following respects: clypeus slightly shorter; frons and vertex more convex; pronotum with anterior angles obtuse and lateral angles rounded; elytra 1.7 times longer than wide; abdomen almost flat; pygidium 1.7 times wider than long; tibiae broader and robust; metafemora slightly broader; and inner metatibial spur wider and longer than metatarsomere 1.

#### Etymology.

The specific epithet is derived from Chiapas, the state of Mexico where this species was collected, combined with the Latin suffix –*ensis*, meaning belonging to.

#### Distribution.

*Diplotaxis chiapasensis* sp. nov. is only known from the type locality, situated on the Pacific side of the state of Chiapas, Mexico, near the border with Guatemala (15°05'6.9"N, 92°05'02.24"W) (Fig. 17). This locality is at 1660 m altitude, with cloud forests with different degrees of disturbance.

#### Taxonomic remarks.

*Diplotaxis chiapasensis* sp. nov. is similar to *D. xalapensis*. Both species belong to the *trapezifera* group because of the setose clypeus and the rest of the dorsum glabrous, but both species can be distinguished from the remaining species of this group by the dorsoventrally flattened body and the elytra dark brown. *Diplotaxis chiapasensis* sp. nov. is clearly separated from *D. xalapensis* by the shiny elytra (not matt or with sericeous surface).

### Diplotaxis complanatis sp. nov.

Taxon classificationAnimaliaBrassicalesBrassicaceae

11B270A8-47F1-53B8-810F-357AAC37D69E

http://zoobank.org/8908C72D-8A47-4A2E-B8B9-3249B5B62155

Figs 11–16

#### Material examined.

***Holotype*** male, “México: Oaxaca, km 21 Carr. Yolotepec-Juquila, VIII-1993, Alt. 1,900 m, bosque mesófilo, luz, L. Delgado col.” (IEXA). ***Paratype*** female, same data as holotype (IEXA). Three male and one female ***paratypes***, same data except: “31-VII–1-VIII-1991, luz u.v., J. L. Navarrete, G. Quiroz y L. Delgado cols.” (CNIN, SMC, LLDC).

#### Diagnosis.

This tiny species is recognized by the following combination of characters: body dorsoventrally flattened, clypeal surface with a few and minute setae, pronotum and elytra glabrous or with scarcely visible setae (shorter than diameter of one puncture), and dorsum shiny but without a metallic cast.

#### Description.

**Figures 11–16.**
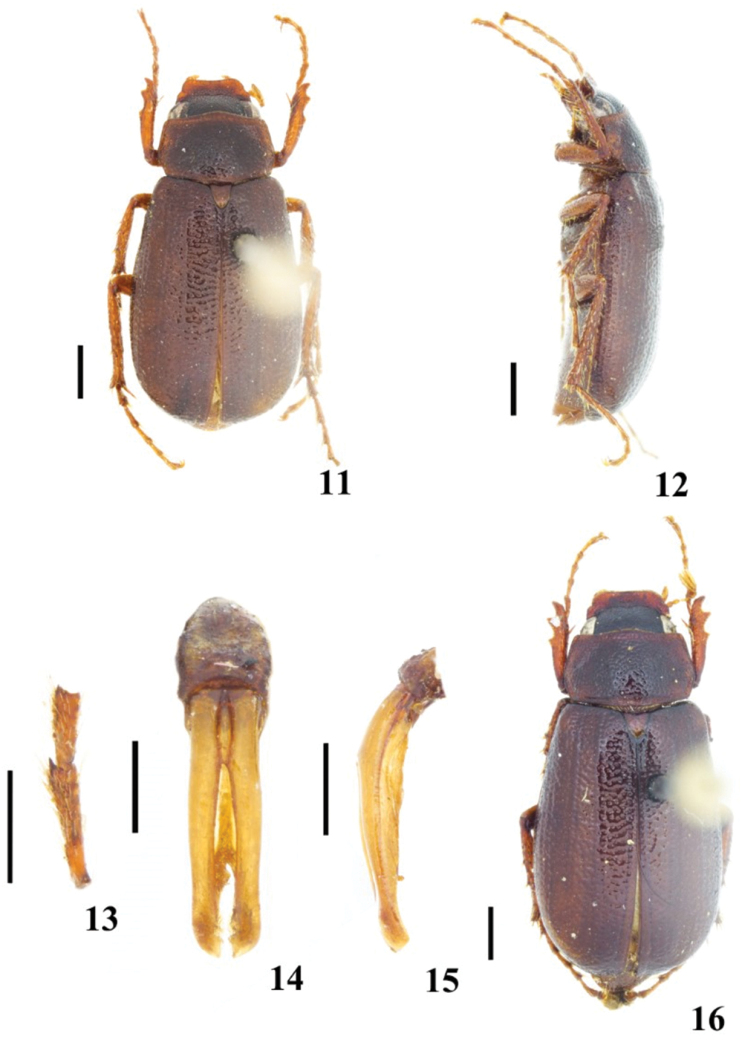
*Diplotaxis complanatis* sp. nov. **11** male habitus, dorsal view 1**2** male habitus, lateral view 1**3** male protarsomeres **14** male genitalia, frontal view **15** male genitalia, lateral view **16** female habitus. Scale bars: 1 mm (**11, 12, 16**); 0.5 mm (**13–15**).

***Holotype*. Male** (Figs 11–15). Length 6.58 mm; width 3.26 mm. Body elongate and dorsoventrally flattened. Clypeus reddish, frons and vertex black, pronotum reddish, elytra reddish-brown; dorsum shiny, without metallic cast.

***Head*.** Clypeus trapezoidal in shape, length equals 0.66 that of frons, apex broadly emarginated with anterior angles rounded, and sides indented in front of eyes; surface concave, coarsely rugose, with scarce, minute setae near external margins; frontoclypeal suture barely marked; frons slightly concave, gradually declivous to clypeus, with large and dense punctures; transverse eye diameter 0.34 interocular width; antennae 10-segmented; labrum with anterior half slightly convex and posterior half concave, length equals 0.50 of that of reflexed underside of clypeus, surface with small, sparse punctures; mandibles moderately robust in frontal view; mentum convex, with anterior declivity marked by an arcuate, setiferous ridge; last article of maxillary palps not impressed dorsally.

***Pronotum*.** Hexagonal in shape, anterior angles right, lateral margins obtusely angled near middle, posterior angles obtuse; surface slightly convex, with a shallow fovea on each side; punctation coarse on disc, confluent along sides; basal margin beaded, with a row of small punctures.

***Scutellum*.** With sparse, medium-sized punctures. ***Elytra***. 1.7 times longer than width, broad intervals with coarse punctures, many of which confluent, narrow intervals slightly raised; elytral punctures with setae minute, barely visible.

***Abdomen*.** Without lateral carina; propygidium without groove anterior to pygidium; ventrites 2–5 subequal in length, surface with small setae; pygidium 1.8 times wider than long, slightly convex in basal 3/4, apical fourth flat; surface with coarse, deep punctures, with sparse setae on apical third.

***Legs*.** Protibiae tridentate, basal tooth situated nearly at middle and removed from apical teeth; claws bent and subapically cleft; tarsi longer than respective tibiae; apex of protarsomere 2 with a small denticle (Fig. 13), mesotarsomere 1 longer than 2; metacoxal plates margined and rounded laterally; metafemora straight and slender; metatibial spurs long and acute; metatarsomere 1 shorter than 2, and almost as long as longest spur.

***Genitalia*.** Basal piece almost as long as parameres, parameres joined along inner margin in basal third, almost parallel, and with apices rounded and slightly widened (Figs 14–15).

#### Variation.

Three male and two female paratypes. Males: length 6.5–7.2 mm, width 3.1–3.3 mm. Females: length 7.2–7.6 mm, width 3.6–3.9. In both sexes, the color and punctation varies slightly. Females differ from males in having frons more convex; abdomen slightly more convex; tibiae and femora broader and robust, protarsomere 2 without a denticle; inner metatibial spur longer than metatarsomere 1.

#### Etymology.

The name of this species is derived from the Latin *complanatae*, meaning flat, in relation to the dorsoventrally flattened body.

#### Distribution.

**Figure 17.**
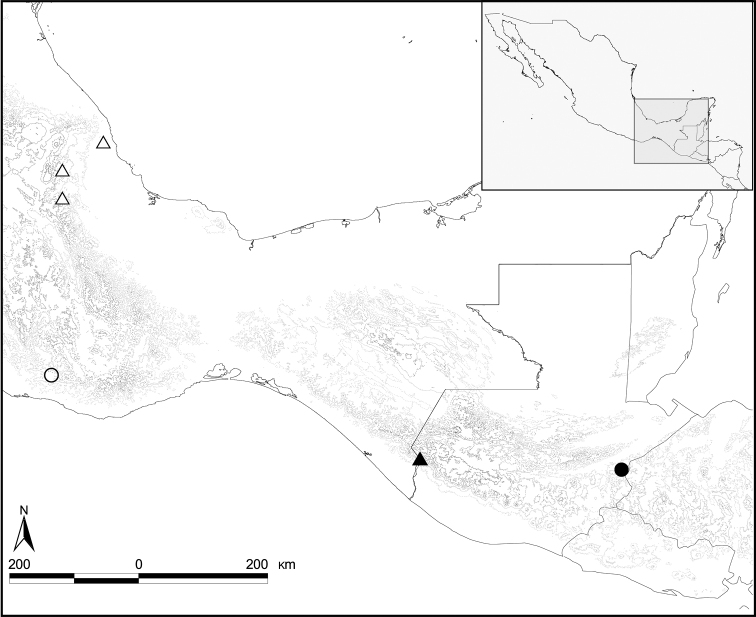
Distribution of *Diplotaxis* species. *Diplotaxis balam* sp. nov. (black circle), *D. chiapasensis* sp. nov. (black triangle), *D. complanatis* sp. nov. (white circle), *D. xalapensis* Delgado and Capistrán (white triangle).

This species is known only from the type locality, which is situated in the Sierra Madre del Sur, in the state of Oaxaca, Mexico (16°14'33.4"N, 97°15'01"W) (Fig. 17). The locality is on the slope facing southward to the coast, at 1900 m altitude, in a transition between pine-oak and cloud forests. The specimens were attracted to ultraviolet light traps.

#### Taxonomic remarks.

This small species has a dimorphic character which is so far unique for this genus: the presence in the males of a minute denticle on the apex of protarsomere 2 (Fig. 13). The clypeus with minute setae and the rest of the dorsum glabrous (or nearly so) relate this species with the *trapezifera* group, however, *D. complanatis* sp. nov. exhibits a dorsoventrally flattened body. This species can be distinguished from *D. chiapasensis* sp. nov. and *D. xalapensis* by its shiny body, but without a metallic green cast (Figs 11, 16).

## Discussion

The three species herein described, in addition to *D. xalapensis*, are distinguished from the other species of the genus *Diplotaxis* by the following combination of characters: body dorsoventrally flattened, clypeal surface with at least a few and distinct setae, and pronotum and elytra glabrous or with scarcely visible setae (most setae shorter than one puncture diameter). The presence of setae on the clypeal surface, together with the pronotum and elytra glabrous or nearly, could include these species in the *trapezifera* species group. The species of this group, however, exhibit a convex body, a character widespread in this genus. Without an analysis of the phylogenetic relationships of the species of this genus (which is currently being carried out by the senior author), the flattened body character state should not, at this time, be considered as a synapomorphy, but only as an uncommon character state.

Besides these species, there are two species showing a flattened body, *D. hallei* Vaurie and *D. pilifera* Burmeister, but these species are included in the *pilifera* group by their distinctive scales on the dorsum and venter (Vaurie 1958). Because the new species here described, along with *D. xalapensis*, share the characters mentioned above with the *trapezifera* group, we included them in this group, and modified Vaurie’s key accordingly (modifications are indicated by a lowercase letter).

### Key to the *trapezifera* group [partly modified from Vaurie (1960)]

**Table:** 

26	Front margin of pronotum at sides drawn forward to acute angle	**27**
26'	Front margin of pronotum at sides truncate or virtually so, forming obtuse or right angle	**27a**
27	Pronotum with sides strongly arcuate behind middle, and hind angles rounded; lateral margins of clypeus almost parallel with indentation in front of eyes	***D. incisa* Vaurie**
27'	Pronotum with sides scarcely arcuate and hind angles distinctly angulate; lateral margins of clypeus without indentation in front of eyes	***D. saltensis* Vaurie (in part)**
27a	Dorsum red or reddish-brown	**27b**
27a'	Dorsum of different color, sometimes with metallic cast	**27d**
27b	Head and pronotum shiny; elytra yellowish, with scattered black foveae	***D. balam* sp. nov.**
27b'	Head and pronotum with metallic green cast; elytra of different color, without black foveae	**27c**
27c	Elytra dull green, sericeous	***D. xalapensis* Delgado & Capistrán**
27c'	Elytra shiny green	***D. chiapasensis* sp. nov.**
27d	Body dorsoventrally flattened; clypeus rectangular, with front angles rounded	***D. complanatis* sp. nov.**
27d'	Body dorsoventrally convex; clypeus trapezoidal, with front angles right or acute	**28**
28	Eyes very large, each eye about 1/3 or nearly of width of head; size small (6 to 7 mm)	**29**
28'	Eyes not quite so large, each 1/5 or 1/4 of width of head; size usually larger than 7 mm	**30**

## Supplementary Material

XML Treatment for Diplotaxis balam

XML Treatment for Diplotaxis chiapasensis

XML Treatment for Diplotaxis complanatis
